# mGem: Immune recognition and clearance of bacteriophages—implications for phage therapy

**DOI:** 10.1128/mbio.01418-25

**Published:** 2025-11-05

**Authors:** H. T. Le, G. Ahlenstiel, C. Venturini, S. A. Read

**Affiliations:** 1 Blacktown Clinical School, Western Sydney University 522514, Sydney, New South Wales, Australia; 2 Storr Liver Centre, Westmead Institute for Medical Research, Sydney, New South Wales, Australia; 3 Blacktown Mt Druitt Hospital, Sydney, New South Wales, Australia; 4 Centre for Infectious Diseases and Microbiology (CIDM), Westmead Institute for Medical Research, Sydney, New South Wales, Australia; 5 Sydney School of Veterinary Science, Faculty of Science, University of Sydney 98482, Sydney, New South Wales, Australia; Albert Einstein College of Medicine, Bronx, New York, USA

**Keywords:** bacteriophage, phage therapy, adaptive immunity, cell-mediated immunity, viral immunity

## Abstract

Bacteriophages (phages) hold significant promise as targeted antibacterial therapies in the era of rising multidrug-resistant infections. Despite their therapeutic potential, the clinical application of phages for human infections has been significantly hindered by the rapid and robust immune response to phages in blood. The rapid clearance of >99% of phages from circulation within hours of injection is the result of innate and adaptive immune responses that target therapeutic phage for clearance and destruction. Methodologies must be developed to isolate and/or modify phages that are not only therapeutically potent but also immunologically camouflaged. The resulting second- and third-generation phage therapies will be more effective by evading host immune responses, enabling more efficient targeting of bacterial pathogens.

## PERSPECTIVE

Bacteriophages (phages) are viruses that exclusively infect bacteria and cannot replicate within eukaryotic cells in the absence of their bacterial host. Discovered in 1915 (1, 2), more than 10 years before penicillin, phages were immediately considered for treating bacterial infections with apparent success in curbing a cholera epidemic. Phage therapy was largely disregarded by most countries following the introduction of antibiotics but has regained clinical relevance in recent decades as a consequence of the escalating antimicrobial resistance crisis, particularly for treatment of multidrug-resistant infections. The safety of phage therapy and potential as adjunct intervention has been proven repeatedly in compassionate cases (3, 4), demonstrating the value for development of phage-based clinical approaches.

Conceptually, phages are an ideal therapeutic antibacterial due to their narrow host range (hence specificity) and potent bactericidal activity. In practice, however, the human immune system employs multiple strategies to entrap, inactivate, and remove phages from circulation, slowing clinical translation. Indeed, intravenous (IV) injection of different phages in mice and humans consistently results in ≥99% removal from blood within 6 hours (5–8). Ultimately, effective phage therapy must mitigate the human immunological response to phages without compromising the host immune response to the primary bacterial infection. Ultimately, synergy between the human immune system and phage (immunophage synergy [9]), as opposed to antagonism, will best improve patient outcomes.

To address this dilemma, we must ask “why” phages are targeted by the human immune system. After all, they pose no “direct” threat to human health. Is there an evolutionary advantage to removing phage particles from circulation? Alternatively, do we possess sweeping immunological processes targeting antigenic motifs that are shared among pathogenic and non-pathogenic microbes alike? Contemporary research suggests that both may be playing a role. Clinical-grade phage preparations that lack bacterial contaminants can elicit potent inflammatory and antiviral responses *in vitro* (10, 11) and *in vivo* (12, 13), indicating that phage antigens can produce potentially harmful responses. A current focus of study, therefore, is the modification of phage particles to dampen antiphage immune responses and prolong the half-life of phages in blood. In doing so, phage-based therapeutics will be not only safer but also more effective by promoting phage-mediated bacterial killing through improved longevity in circulation.

## IMMUNE-MEDIATED PHAGE CLEARANCE

**Fig 1:**
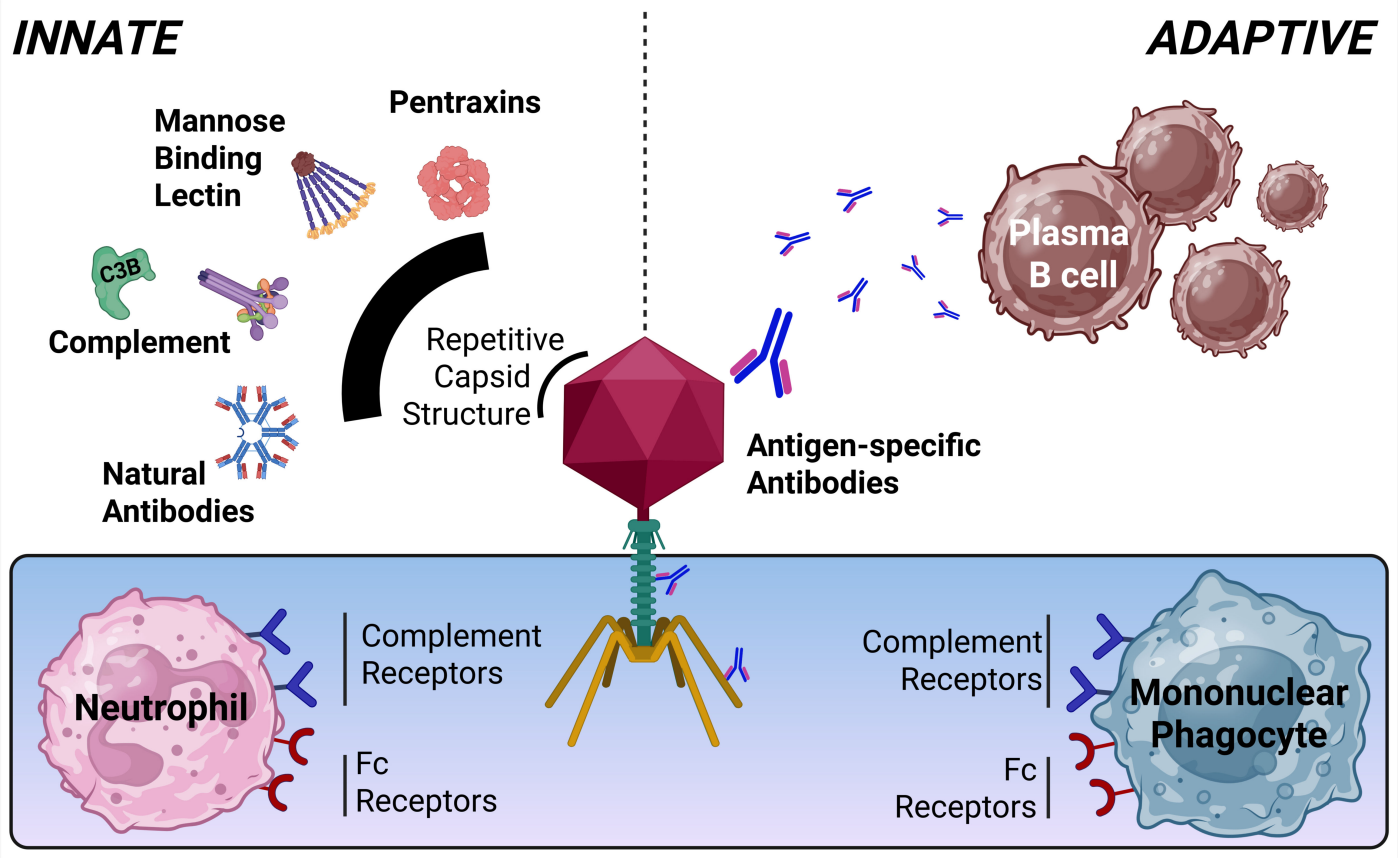
Innate and adaptive immune recognition of phage particles. Upon IV administration of phages, soluble innate immune proteins, including natural antibodies, mannose-binding lectin (MBL), and acute-phase response proteins such as complement and pentraxins may bind phage particles to opsonize and impair phage infectivity. This has not been demonstrated experimentally for MBL and pentraxins but is a strong possibility due to the heteromeric composition of innate immune proteins and repetitive capsid structure of phages. Combined with the production of adaptive antigen-specific antibodies, phagocytic cell populations in the blood and tissue recognize and engulf opsonized phage via complement and antibody Fc receptors.

IV phage administration enables systemic delivery and achieves higher bioavailability compared to oral, nasal, or topical routes (14). Once in circulation, however, rapid clearance of phage is coupled with accumulation primarily in the liver (15, 16), followed by the spleen (15, 17, 18), both organs containing abundant phagocytic immune cell populations. In addition to circulating phagocytes such as monocytes and neutrophils (10), splenic macrophages and Kupffer cells (liver macrophages) are primary drivers of phage engulfment (17, 19). As one might expect, macrophage depletion can significantly increase phage duration in circulation (20). Intriguingly, however, B-cell depletion also significantly impairs clearance following IV T7 phage administration (21), suggesting that both innate and adaptive immune systems work in concert to clear phages from blood. These data indicate that opsonization (the coating of foreign particles with molecules to increase immune recognition and phagocytosis) is essential to immune-mediated phage clearance. Opsonins include antibodies, complement proteins, and lesser-known pattern recognition molecules such as mannose-binding lectin (MBL) and pentraxins (Fig. 1). Phagocytes, such as neutrophils, macrophages, and dendritic cells, express receptors to recognize opsonins (e.g., antibody Fc receptor CD16 and complement receptors) that promote the capture and internalization of opsonized material (22).

### Antibodies

In both mouse and human models, phage administration stimulates antibody production (23, 24) that promotes phage clearance (25), neutralizes phage infection *in vitro* (26, 27), and impacts therapeutic efficacy (28). The antigenic targets of these antibodies are diverse, polyclonal, and directed against phage structural proteins, including the major capsid and receptor-binding proteins, as well as decorative proteins like Hoc and Soc (29–31). In addition to phage therapy-driven antibody production, phage-neutralizing antibodies are present in up to 40% of individuals who have not undergone phage therapy (32). This finding supports the presence of either (i) cross-reactive antibodies generated against environmental phages or (ii) phage neutralization by natural antibodies. Natural antibodies are low-affinity, polyreactive antibodies generated by B cells without a simulating antigen (33) and bind evolutionarily conserved epitopes occurring in microbes such as phospholipid phosphorylcholine (34) or glycans (35). Foreseeable improvements to current phage therapy pipelines may therefore include the quantification of baseline and treatment-induced neutralizing antibodies (at a patient-specific and community level). While these data may provide important insights into phage immunogenicity, the relationship between antibodies and therapeutic efficacy remains uncertain, as the development of antiphage antibodies does not exclude positive clinical outcomes (36).

### Complement

In addition to antibodies, complement deposition on phage has been well documented, inhibiting phage infectivity (37, 38) and acting as an opsonin to increase phagocyte engulfment (10). Importantly, complement deposition initiated by both natural IgM antibodies (39) and acquired phage-specific antibodies (30) via the classical pathway of complement activation has been documented. Phage-mediated complement pathway activation by other innate signaling molecules has not been shown but is possible. Soluble heteromeric innate sensors such as IgM, MBL, and pentraxins function to recognize repetitive protein motifs such as those found in phage capsids (40) (Fig. 1) and may stimulate the deposition of complement. Pentameric C-reactive protein and serum amyloid P are prime examples of pentraxins secreted by the liver as part of the acute-phase response that are deposited on microbes and dying cells, facilitating the binding of complement factors (41).

In summary, it may be just as important to routinely measure antiphage immune responses (e.g*.,* natural and acquired antibodies), in addition to circulating phage concentration during phage therapy. These data will provide insights into the mechanisms and antigens that trigger phage recognition, aiding the development of second- and third-generation phage therapies based on modified phages and/or immunomodulation.

## PHAGE CLEARANCE VIA NON-IMMUNE MECHANISMS

Phage uptake is not restricted to immune cells. Upon intravenous injection, circulating phages also interact with the blood vessel endothelium, which must be transversed to enter tissue. This may be desirable to reach a site of infection but requires transcytosis of phage as opposed to engulfment and intracellular degradation. T4 phage transcytosis has been documented across endothelial and epithelial cell lines (42) but has also been shown to accumulate within cell monolayers where they become largely inactivated (43).

The liver is the primary site of phage accumulation *in vivo*, where both Kupffer cells and, to a lesser degree, primary liver sinusoidal endothelial cells (LSECs) filter phage from blood (19). LSEC internalization and degradation of K1F and T4 phage have been demonstrated *in vivo* (19) and *ex vivo* (44), respectively, in a manner that appears independent of known LSEC endocytosis receptors stabilin 1/2, mannose receptor, or the FcγRIIb2. In line with immune-mediated internalization, understanding the mechanisms of non-immune internalization will be important to prevent phage clearance and, if needed, promote transcytosis into tissues. Indeed, interactions with cell surface receptors may be key to this process. Studies using cancer cell lines have shown that phages can bind sialic acid (SA) glycans (45) and integrins (46). Interestingly, this represents a shared mechanism of internalization among prokaryotic and eukaryotic viruses (47). Multiple low-affinity interactions between repeating icosahedral capsid proteins and highly expressed outer membrane SA/integrin molecules may mediate binding and internalization, which can be inhibited by blocking the interaction (45, 46).

## PHAGE MODIFICATION TO ESCAPE IMMUNE CLEARANCE

The success of phage therapy will require the selection or production of phages that are immunologically camouflaged, extending their duration in circulation to enable bacterial infection and lysis. Unfortunately, as phages are incredibly diverse genetically with minimal characterization of their protein encoding genes, we still know very little regarding phage immunogenicity across the spectrum of phage species. It remains to be determined which structural components of phages lead to their immunogenic properties and whether phage immunogenicity might be predictable via genome characterization.

Phage display may prove to be a particularly useful tool for improving our understanding of the interaction between phage components and the immune system. This method, based on the construction of a polypeptide library fused to phage capsid proteins and affinity selection of binders, has been employed to identify peptide-receptor binding for a variety of applications (48). Phage display can be used to characterize the phage sites that are recognized by the immune system to be targeted for modification, e.g., sites to which antibodies bind (49), and peptides prolonging blood circulation (50) or involved in phage internalization by eukaryotic cells (51) or translocation to body sites (52).

Both genetic engineering (e.g., recombineering or CRISPR-Cas [53, 54]) and natural techniques (e.g., serial co-incubation and mutant selection [55, 56]) have been explored to reduce phage immunogenicity. Generated phages possess modifications in immunogenic epitopes (e.g., capsid proteins) to prolong phage persistence in blood and reduce the endotoxin load released by bacterial cell lysis (e.g., lethal lysis-deficient phage variants [57, 58]), thus limiting immune activation. In principle, capsid genes encoding proteins that mimic human proteins could also be used to reduce the likelihood of immune recognition. Whether this is feasible is not yet known. Indeed, more work is needed to understand phage epitopes and immunogenic properties in bacterial hosts and humans before any of these approaches can be fully realized.

Cloaking approaches such as liposomes (59) offer immune protection and can lead to controlled phage release into tissues to target intracellular pathogens. Phages targeting extracellular bacteria, however, will likely require modifications to their structural proteins to prevent uptake. While natural capsid glycosylation can impair phage-specific antibody recognition (60), the addition of glycans can drastically increase cellular uptake in a glycan- and organ-specific manner, suggesting that glycosidase treatments may enhance circulation time (61). Additionally, while increasing the positive charge of phage surface proteins via nanocapping has been shown to increase phage engulfment (62), reducing the positive charge can effectively increase the circulating half-life of phage particles (63). This is a particularly enticing avenue for research as it aligns with long-circulating lambda phage mutants that possess an acidic glutamic acid residue in place of the wild-type basic lysine residue (56). Lastly, coating of phage capsids with polyethylene glycol has also shown to prolong phage circulation and reduce immune recognition, representing another phage modification option (64).

## CONCLUSION

While phages offer advantages such as host specificity and natural abundance, their clinical utility appears to be limited by rapid immune clearance and immunogenicity. Genetic engineering, encapsulation, and phage immunogenicity screening approaches have shown potential to enhance phage stability and prolong circulation. Whether these approaches will truly improve the therapeutic effect of phage therapies *in vivo* remains to be seen. Nonetheless, the first successful use of an engineered phage created via bacteriophage recombineering of electroporated DNA against drug-resistant *Mycobacterium abscessus* highlights the translational potential of such strategies (65). To fully realize the potential of phage therapy, future work must integrate immunological insights, synthetic biology, and targeted delivery to create durable, effective, and personalized treatments.
